# Ozone oil promotes wound healing by increasing the migration of fibroblasts via PI3K/Akt/mTOR signaling pathway

**DOI:** 10.1042/BSR20170658

**Published:** 2017-11-09

**Authors:** Weirong Xiao, Hua Tang, Meng Wu, Yangying Liao, Ke Li, Lan Li, Xiaopeng Xu

**Affiliations:** Dermatological Department, Hunan Provincial People’s Hospital & The First Affiliated Hospital of Hunan Normal University, Changsha 410005, P.R. China

**Keywords:** EMT, fibroblasts, ozone oil, wound healing

## Abstract

Skin injury affects millions of people via the uncontrolled inflammation and infection. Many cellular components including fibroblasts and signaling pathways such as transforming growth factor-β (TGF-β) were activated to facilitate the wound healing to repair injured tissues. C57BL/6 female mice were divided into control and ozone oil treated groups. Excisional wounds were made on the dorsal skin and the fibroblasts were isolated from granulation tissues. The skin injured mouse model revealed that ozone oil could significantly decrease the wound area and accelerate wound healing compared with control group. QPCR and Western blotting assays showed that ozone oil up-regulated *collagen I, α-SMA*, and *TGF-β1* mRNA and protein levels in fibroblasts. Wound healing assay demonstrated that ozone oil could increase the migration of fibroblasts. Western blotting assay demonstrated that ozone oil increased the epithelial–mesenchymal transition (EMT) process in fibroblasts via up-regulating fibronectin, vimentin, N-cadherin, MMP-2, MMP-9, insulin-like growth factor binding protein (IGFBP)-3, IGFBP5, and IGFBP6, and decreasing epithelial protein E-cadherin and cellular senescence marker p16 expression. Mechanistically, Western blotting assay revealed that ozone oil increased the phosphorylation of PI3K, Akt, and mTOR to regulate the EMT process, while inhibition of PI3K reversed this effect of ozone oil. At last, the results from Cytometric Bead Array (CBA) demonstrated ozone oil significantly decreased the inflammation in fibroblasts. Our results demonstrated that ozone oil facilitated the wound healing via increasing fibroblast migration and EMT process via PI3K/Akt/mTOR signaling pathway *in vivo* and *in vitro*. The cellular and molecular mechanisms we found here may provide new therapeutic targets for the treatment of skin injury.

## Introduction

Wound is caused by trauma, burn, ulcer, surgery, and others. Although most of the wounds generally heal well, the failure of wound healing affects millions of people in the world through the uncontrolled inflammation and infection [1,2]. Wound healing is composed of many complex processes which include inflammation response, new tissue formation, and tissue remodeling [1,3]. In the first 48 h after injury, different immune cells such as neutrophils, monocytes, and lymphocytes work together to prevent the bleeding and remove the dead tissues to balance the inflammation process and make the appropriate repair of the wound [4,5]. In the next 2–10 days, new tissue formation is followed via cellular proliferation and migration of different cell types such as fibroblasts, keratinocytes, and endothelial cells. At this stage, fibroblasts play very important roles in the new tissue formation [2]. The wound will attract amount of fibroblasts to the injury sites to facilitate the wound healing via different mechanisms [6,7]. For example, wound production can increase the proliferation and migration of fibroblasts to promote the scar formation [8,9]. In addition, fibroblasts can secrete many factors, such as matrix metallopeptidase-14 (MMP-14), basic fibroblast growth factor (bFGF), fibroblast growth factor-9 (FGF-9) to regulate the collagen homeostasis, angiogenesis, or other important functions to facilitate the wound healing [10-13]. Also, fibroblasts can differentiate into myofibroblasts [14], which produces extracellular matrix and ultimately forms the mature scar [15]. In 2–3 weeks after injury, the tissue remodeling process happens which may last for a year or more. At this stage, the entire processes activated by injury will wind down and cease while the activated cells will undergo apoptosis. Different cells (fibroblasts, macrophages, and endothelial cells) will secrete matrix metallopeptidase to remodel and strengthen the repaired tissues [1,16]. Through these classic wound healing processes, the wound will be repaired.

Ozone, made up of three oxygen atoms, is a natural gaseous molecule which can be in gaseous and liquid forms. Ozone can react with blood components to affect oxygen metabolism, antioxidant defense system, cell energy, and microcirculation [17]. Furthermore, ozone can activate the expression of many cytokines which are important for wound healing, and antibacterial and antiviral activity [18]. Thus, ozone therapy (OT) has been widely used for improving wound healing, as an antibacterial agent, and modulating immune system [19,20]. In the treatment of tissue injury, OT is getting more and more attention. For example, OT can improve the wound healing via enhancing blood perfusion [17]. And OT induces the expression of vascular endothelial growth factor (VEGF), transforming growth factor-β (TGF-β), and platelet-derived growth factor (PDGF) to facilitate wound healing of diabetic foot ulcers [21]. OT also has been applied for the clinical treatment of tissue injury and has shown very good results [22,23]. But till now, no research focusses on the effect of ozone oil, which is produced via bubbling high concentrations of ozone in the oil to retain the ozone, on wound healing, and the underlying mechanisms’ study.

Epithelial–mesenchymal transition (EMT) is an important physiological process during embryogenesis and tumorigenesis [24,25]. EMT is characterized by loss of epithelial proteins, such as E-cadherin, and increase in several mesenchymal proteins, such as vimentin, fibronectin, and N-cadherin [24,26]. Many kinds of factors can trigger the EMT process. Amongst these factors, TGF-β plays important roles in EMT via diverse downstream pathways, like Smads, RhoA, MAPK, and PI3K [27-30]. Recently, activation of PI3K/Akt/mTOR signaling pathway by TGF-β is critical for the regulation of EMT [31-33]. The activation of PI3K can activate mTOR via Akt to accelerate the EMT. Although many studies have found that EMT plays critical roles in cancer development [31,33], EMT has been proved to participate in the wound healing [34,35]. For example, impairment of fibroblasts’ growth and EMT in vinmentin or slug-deficient mice demonstrate to slow the wound healing [36,37]. Tumor necrosis factor-α (TNF-α) can promote the fibroblast EMT process via induction of bone morphogen protein (BMP) 2/4 to facilitate the wound healing [38]. However, whether ozone oil can promote the wound healing via regulating EMT process through PI3K/Akt/mTOR signaling pathway is still unknown.

At present, the functional study of the ozone oil drug on wound healing is still lacking and the underlying mechanisms are still unknown. Furthermore, no studies have been carried out to elucidate if ozone oil can promote the wound healing via increasing the migration of fibroblasts. In our study, we found that the ozone oil drugs facilitate the wound healing through promoting the migration of fibroblast via activating PI3K/Akt/mTOR signaling pathway *in vitro* and *in vivo* studies.

## Materials and methods

### Mice model for wound

C57BL/6 female mice (7 weeks old, *n*=24) were separated for two groups, control and ozone oil treated groups. Ozone oil, which contains 99% ozonide, superoxide, and *Camellia* oil, was bought from Healthcare Technology, Inc, China. The mice were first anesthetized with chloral hydrate followed by the shaving of the area assigned for wounding under sterile conditions. Excisional wounds with 1-cm diameter were created on the dorsal skin on day 0. Then the mice were maintained in sterile conditions and ozone oil was applied for the treatment from day 1. The treatment of wounded skin with 400 μl ozone oil was done with cotton swabs for 12 days (once every 2 days) compared with the control group without ozone oil. The areas were measured every 2 days to evaluate the therapeutic efficiency. All the procedures involving animals and their care in the present study were performed in accordance with the Guidelines for the Care and Use of Laboratory Animals of Provincial People’s Hospital in Hunan.

### Isolation and culture of fibroblasts from new tissues

The granulation tissues were isolated from dorsal skin of the injured mice which were cut into 1 mm^3^ by scissors under sterile conditions. The tissues were digested with 0.25% trypsin (25200056, Gibco) for approximately 60 min at 37°C. Then, DMEM medium (11995065, Gibco) containing 10% FBS (16000044, Gibco) was added to stop the digestion. The solution was passed through 70-μm filters (352360, BD Falcon) to remove the undigested tissues and large cell aggregates followed by the centrifugation at 1500 rpm for 5 min. The cell pellet was washed twice with DMEM medium and suspended in DMEM medium containing 10% FBS and seeded into 12-well plates (3335, Corning). They were incubated in a humidified incubator with an atmosphere of 95% air and 5% CO_2_ at 37°C. The cultured fibroblasts were further treated with ozone oil *in vitro*. For every well, 10 μl ozone oil was used for the treatment of cultured cells. For the treatment of LPS, the cultured fibroblasts were treated with 100 ng/ml LPS with or without ozone oil for 24 h. For the treatment of PI3K inhibitor, LY294002 (L9908, Sigma), the cultured fibroblasts were pretreated with 10 μM LY294002 then treated with ozone oil for 24 h.

### Wound healing assay

For the migration assay of fibroblasts, the fibroblasts were isolated and cultured from the granulation tissues of injured skin in ozone oil treated and control groups. The fibroblast monolayers were carefully scratched using a 10-μl pipette tip. Then, the cells were further treated with or without ozone oil for 24 h. After 24 h, the wounded area was photographed. The empty area which indicates the wound region was calculated.

### Cytometric bead array

The injured fibroblasts isolated from the mice were plated in 24-well plates with 4 × 10^4^ cells/well (0.5 ml). After incubation overnight, the fibroblasts were pretreated for stimulation with LPS (100 ng/ml) with or without ozone oil for 24 h. Cytokines in the culture medium were quantitatively measured by Cytometric Bead Array (CBA) human inflammation kit (BD Biosciences, San Jose, CA, U.S.A.) according to the manufacturer’s instructions. Data were acquired using CELLQuest software on the flow cytometer (FACSCalibur; BD Biosciences). The CBA kit can detect two cytokines including IL-6 and TNF-α with a minimum detectable level of 5 pg/ml.

### Real-time PCR

Total RNA was extracted from cultured fibroblasts treated with or without ozone oil by TRIzol (15596018, Invitrogen) according to the manufacturer’s instructions. cDNA was synthesized from total RNA with the First-strand Synthesis System (2680A, Takara) according to the manufacturer’s instructions. Real-time PCR was performed by SYBR Premix Ex Taq II (DRR081A, Takara) with 7500 Real-Time PCR system (Applied Biosystems) and normalized to the expression of GAPDH. Relative quantitation and statistics were estimated as the mean of three replicate assays calculated by the 7500 FAST system sequence detection software Q17 (Applied Biosystems). The primers used are:

- Collagen I-F: 5ʹ-GCTCCTCTTAGGGGCCACT-3ʹ
- Collagen I-R: 5ʹ-CCACGTCTCACCATTGGGG-3ʹ
- α-SMA-F: 5ʹ-AGGGAGTAATGGTTGGA ATGG-3ʹ
- α-SMA-R: 5ʹ-GGTGATGATGCCGTGTTCTA-3ʹ
- TGF-β1-F: 5ʹ-CTCCCGTGGCTTCTAGTGC-3ʹ
- TGF-β1-R: 5ʹ-GCCTTAGTTTGGACAGGATCTG-3ʹ
- Gapdh-F: 5ʹ-AGGTCGGTGTGAACGGATTTG-3ʹ
- Gapdh-R: 5ʹ-TGTAGACCATGTAGTTGAGGTCA-3ʹ.

### Western blotting

Cells were lysed in RIPA buffer on ice. After centrifugation at 12000***g*** for 15 min at 4°C, the supernatant was collected and the concentration was measured using BCA method. Fifty micrograms of total protein were separated by SDS/PAGE. Then, proteins were transferred on to PVDF membranes (Millipore). After blocking with TBST containing 5% milk for 1 h at room temperature, the membranes were incubated with primary antibodies at 4°C overnight. Next day, the membranes were washed with TBST four times. Then, the membranes were incubated with horseradish peroxidase–conjugated secondary antibody (Santa Cruz Biotechnology) for 1 h at room temperature. The blots were developed by ECL detection reagents (GE Healthcare). The gray intensity of protein bands was quantitated with ImageJ and normalized to GAPDH. The MMP2, MMP9, collagen I, fibronectin, p-Akt (Thr^308^), Akt, p-PI3K (Tyr^458^), PI3K, p-mTOR (Ser^2481^), mTOR, GAPDH antibodies were purchased from Cell Signaling. Insulin-like growth factor binding protein (IGFBP)3, IGFBP5, and IGFBP6 antibodies were purchased from Abcam. α-SMA, N-cadherin, E-cadherin, and p16 antibodies were purchased from BD Biosciences, and vimentin was purchased from Covance.

### Statistical analysis

All data were presented as mean ± S.D. The difference between two groups was analyzed by unpaired two-tailed Student’s *t*test. For multiple comparisons, the one-way ANOVA was used to analyze the difference. The significance of all data was calculated with GraphPad Prism 5.0 software. A value of *P*<0.05 was considered significant.

## Results

### Ozone oil promoted the wound healing in mice

**Figure 1:**
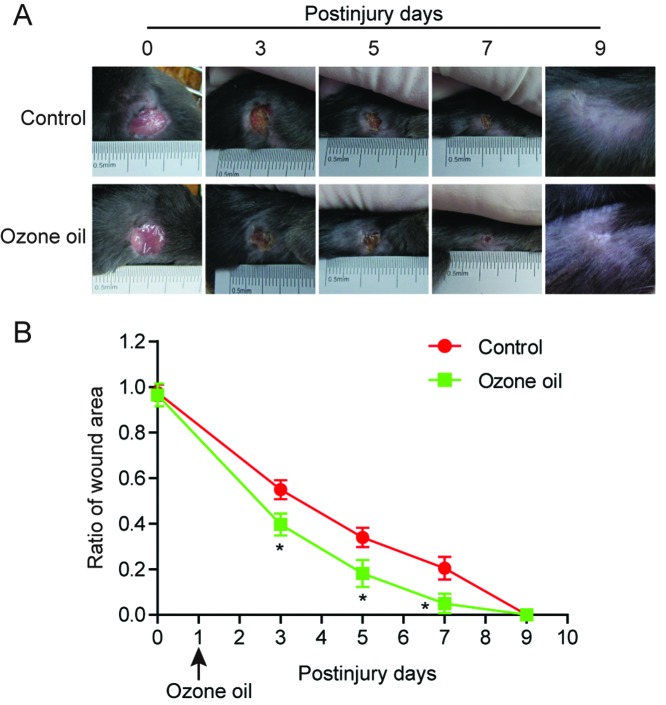
Ozone oil promoted wound healing in a time-dependent manner (**A**) Representative images showing the treatment of ozone oil promoting the wound healing in an injured mouse model. (**B**) The statistical results showing the wound area gradually decreased in a time-dependent manner which was accelerated by ozone oil. Excisional wounds with 1-cm diameter were created on day 0. The ozone oil was applied for the treatment from the day 1. Error bars represent the mean ± S.D.; *P<0.05 compared with control group, respectively.

Previous reports demonstrated that OT could promote the wound healing. To examine if the ozone oil drug can facilitate the mice wound healing, we created a skin injury model in mouse on day 0 (see the ‘Materials and methods’ section) and treated the mouse with ozone oil drug from the next day. The ozone oil was applied to the mice every 2 days. The wound areas were measured every 2 days which lasted for 12 days. We found the wound areas gradually decreased in the control group after injury and the wound areas in ozone oil treated mice decreased much faster than the control group (Figure 1A). Furthermore, the recovery efficiency was much higher and showed time-dependent pattern as ozone oil drug completely cured the skin injury (Figure 1B). These data demonstrated ozone oil drug could significantly promote wound healing in a time-dependent manner.

### Ozone oil activated the fibroblast relevant genes

**Figure 2:**
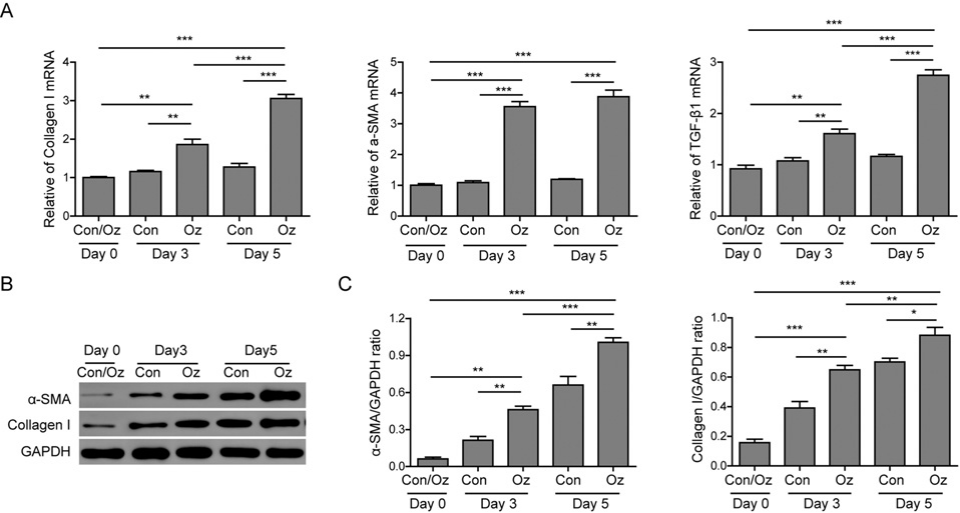
Ozone oil promoted the expression of fibroblast genes (**A**) Real-time PCR results showing that ozone oil increases the fibroblast marker genes, *collagen I, α-SMA*, and *TGF-β1* after injury in a time-dependent manner. (**B**,**C**) Western blotting results showthat the protein expression of these fibroblast genes is also increased after injury. Ozone oil further increases the expression of these genes. Error bars represent the mean ± S.D.; *P<0.05, **P<0.01, and ***P<0.01.

When an injury happens, fibroblasts are activated to promote the wound healing. To elucidate whether fibroblasts participate in the effect of ozone oil drug on wound healing, we isolated and cultured the fibroblasts from the granulation tissues in new tissues at different times. Then we examined the expression of fibroblast relevant genes which are important for the activity of fibroblasts via QPCR and Western blotting. We, first, examined the expression of several fibroblast genes: *collagen-I*, α*-SMA*, and *TGF-β1* at postinjury days 0, 3 and 5. We found that ozone oil treatment increased the expression of these genes in the same time point compared with the control group in a time-dependent manner, although the expression of these genes was not obviously changed in the control group (Figure 2A). Consistent with the mRNA expression results, the protein levels of these genes were also increased after injury and further up-regulated by ozone oil at the same time point compared with the control group (Figure 2B,C). These data demonstrated that ozone oil could promote the wound healing by activating fibroblasts and the fibroblasts activities were gradually increased during the initial stage of new tissue formation.

### Ozone oil promoted the migration of fibroblasts

**Figure 3:**
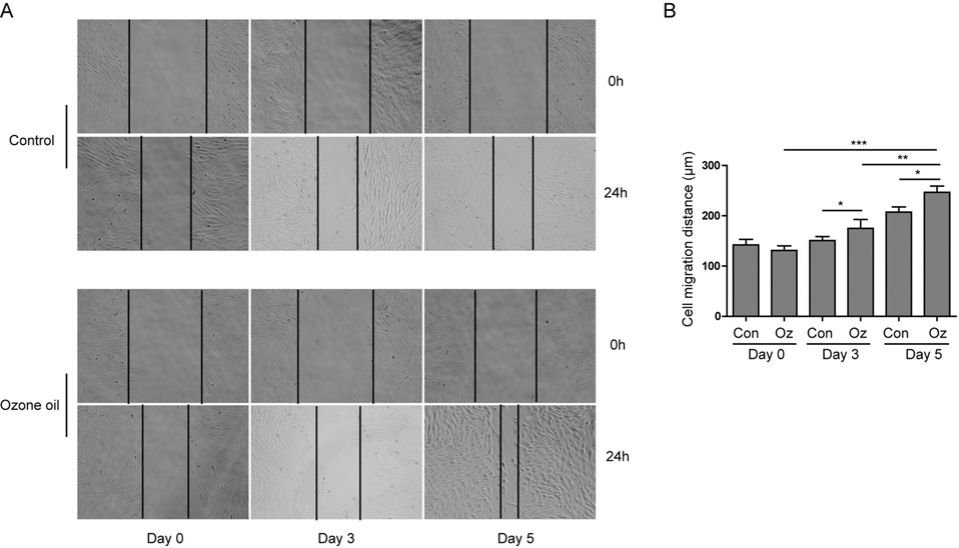
Ozone oil promoted the migration of fibroblasts (**A**) Scratching experiment shows that ozone oil increases the migration of fibroblasts elicited by injury in a time-dependent manner compared with the control group. (**B**) The summary of cell migration distance of fibroblasts isolated from the injury sites of mice with or without the treatment of ozone oil. Error bars represent the mean ± S.D.; *P<0.05, **P<0.01, ***P<0.001.

Next, we wanted to examine the functional effect of ozone oil on the fibroblasts since the migration of fibroblasts is important for wound healing. The classical wound healing assay was applied to examine if ozone oil can affect the migration of fibroblasts. We isolated the fibroblasts from granulation tissues of injured mice at postinjury days 0, 3, and 5 and cultured to form the confluent monolayer. Then, we scratched the confluent fibroblast monolayers to produce a space for the migration of fibroblasts and further cultured the fibroblasts for 24 h followed by the calculation of wound areas. Through the wound healing assay, we found that ozone oil significantly increased the migration of fibroblast compared with the control groups (Figure 3A,B). Furthermore, we found that ozone oil further increased the migration of fibroblasts over the time course of treatment (Figure 3A,B). These data demonstrated that ozone oil could increase the migration of fibroblasts which were elicited by wound in a time-dependent manner.

### Ozone oil up-regulated the EMT process

**Figure 4:**
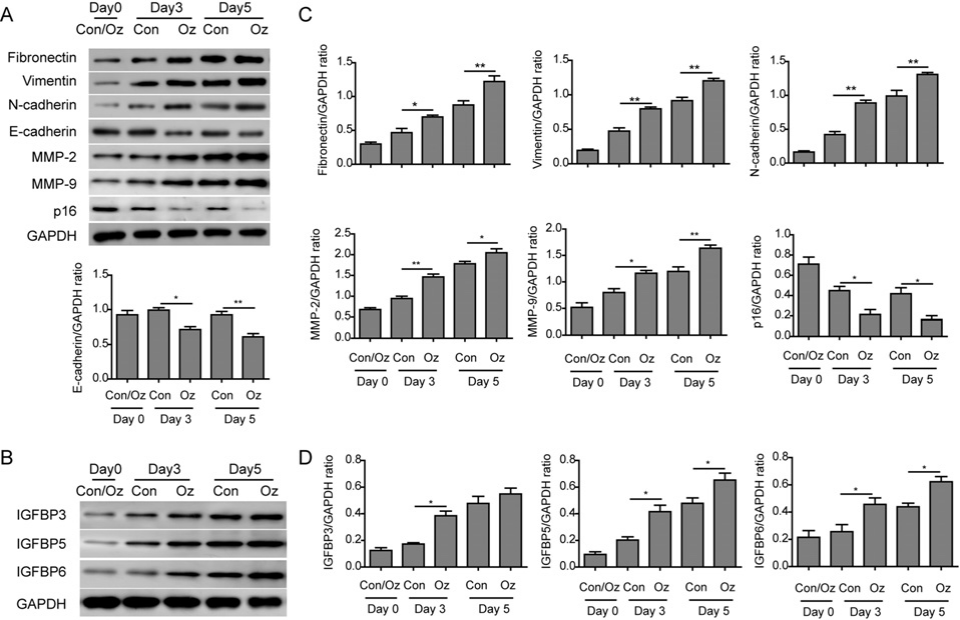
Ozone oil increased the expression of fibroblast EMT proteins (**A**) Western blotting results show that the expression of EMT proteins, fibronectin, vimentin, N-cadherin, MMP-2, and MMP-9 is increased in fibroblasts and further increased by ozone oil in a time-dependent manner, while the epithelial marker (E-cadherin) and cellular senescence marker (p16) are decreased. (**B**) Western blotting results show that the expression of IGFBP3, 5, and 6 increased in fibroblasts and further increased by ozone oil in a time-dependent manner. (**C**) Summary of the Western blotting results in (A). (**D**) Summary of the Western blotting results in (B). Error bars represent the mean ± S.D.; *P<0.05 and **P<0.01.

The results from scratching experiments suggested that ozone oil can promote the migration of fibroblasts. Because the EMT process plays important role in the migration and function of fibroblasts, thus, we next examined if injury can affect the EMT process in the fibroblasts at protein level. By Western blotting assay, we found that the expression of EMT critical proteins fibronectin, vimentin, and N-cadherin were gradually up-regulated in the fibroblasts during the wound healing in a time-dependent manner, while the epithelial protein E-cadherin was not obviously changed during the wound healing in control cells (Figure 4A,C). Importantly, we found the treatment of ozone oil significantly further increased the EMT proteins (Figure 4A,C). Although the expression of E-cadherin was not changed in control group, treatment of ozone oil decreased it at the same point compared with the control group (Figure 4A,C). Furthermore, the expression of matrix metallopeptidase 2 and 9 (MMP2 and 9), which were important for the progression of EMT, were also up-regulated after injury. And ozone oil further increased the expression of MMP2 and MMP9 compared with the control group (Figure 4A,C). Cellular senescence is an irreversible state of cellular arrest which leads to many cellular changes, like protein aggregation, mitochondrial dysfunction etc [39]. The most important molecular mechanism of senescence is p16 and p53 tumor suppressors. Previous studies demonstrate that overexpression of p16 represses fibroblast migration [40,41]. So we examined if the senescence-related gene *p16* participated in the wound healing. We found the expression of p16 after injury was decreased while ozone oil further decreased its expression, which was consistent with previous results (Figure 4A,C). These data indicate that ozone oil can further increase the wound healing via promoting the EMT process.

Previous studies have found IGFBP, which contains six family members and regulates the insulin signaling via binding to IGF, can regulate the EMT process via affecting the TGF-β signaling pathway. Amongst these IGFBPs, IGFBP3, 5, and 6 were extensively studied and had been proven to participate in the EMT process. To prove that EMT process was activated after injury, we also examined the expression of IGFBPs. We found that the expression of IGFBP3, 5, and 6 were significantly increased after injury. And the treatment of ozone oil further increased their expression compared with control group in a time-dependent manner (Figure 4B,D). These results showed that injury elicited the EMT process of fibroblasts and ozone oil can accelerate this process to facilitate the wound healing. Collectively, ozone oil accelerated the fibroblast EMT process via up-regulating the mesenchymal proteins and decreasing the epithelial proteins.

### Ozone oil activated PI3K/Akt/mTOR signaling pathway to promote EMT

**Figure 5:**
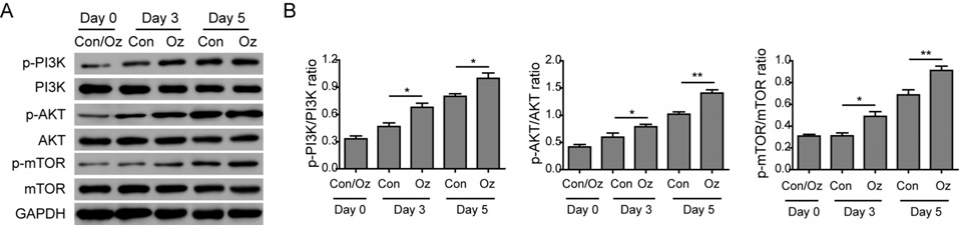
Ozone oil activated the PI3K/Akt/mTOR signaling pathway (**A**) Western blotting results show that the expression of important proteins for PI3K/Akt/mTOR signaling pathway is increased in the fibroblasts. Ozone oil further increases the expression of these proteins. (**B**) Summary of Western blotting results. Error bars represent the mean ± S.D.; *P<0.05 and **P<0.01.

**Figure 6:**
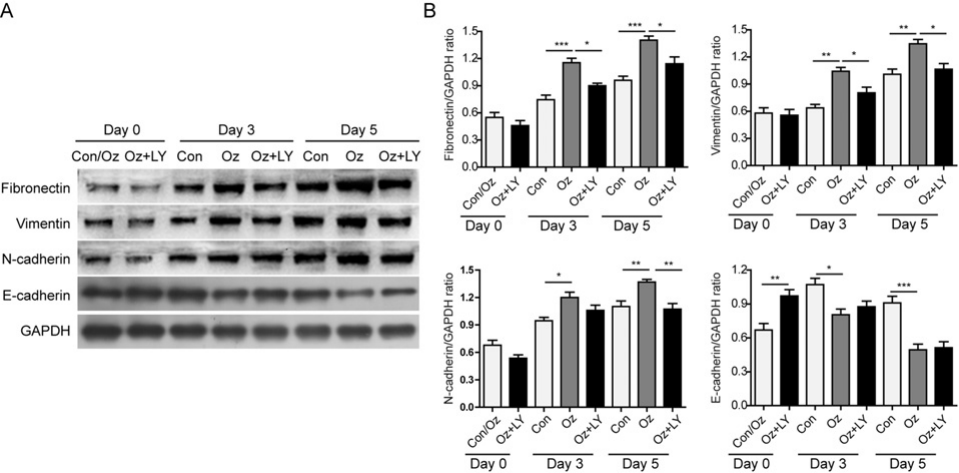
Inhibition of PI3K diminished the effect of ozone oil on the expression of EMT-related proteins (**A**) Fibroblasts isolated from injured mice at 0, 3, and 5 days postinjury were treated with ozone oil with or without PI3K inhibitor, LY294002. The increased expression of fibronectin, vimentin, and N-cadherin, and the decreased expression of E-cadherin after treatment of ozone oil were reversed by LY294002. (**B**) Summary of Western blotting results. Error bars represent the mean ± S.D.; *P<0.05, **P<0.01, and ***P<0.001.

TGF-β is an important inducer of EMT which can activate PI3K. The activation of PI3K can activate Akt and mTOR to accelerate the EMT process. Thus, we examined if the PI3K/Akt/mTOR signaling pathway was involved in the up-regulation of EMT. We isolated the fibroblasts and detected the changes in this pathway via Western blotting. The results showed that the activities of PI3K, Akt, and mTOR were up-regulated after injury because the phosphorylation of these proteins was increased in a time-dependent manner (Figure 5A,B). Consistent with our hypothesis, we found ozone oil treatment further increased the phosphorylation of PI3K, Akt, and mTOR proteins compared with the control group (Figure 5A,B). Then to elucidate if the PI3K/Akt/mTOR signaling pathway is necessary for the injury-induced EMT process, we treated the fibroblasts with ozone oil with or without PI3K inhibitor, LY294002. We found that ozone oil significantly decreased the epithelial marker, E-cadherin, which was consistent with our data (Figure 4A,C), while LY294002 reversed the increased expression of fibronectin, vimentin, and N-cadherin in a time-dependent manner which suggested that PI3K was necessary for the injury-induced EMT process (Figure 6A,B). These data demonstrated that ozone oil accelerated the EMT process via increasing the activity of PI3K/Akt/mTOR signaling pathway in a time-dependent manner.

### Ozone oil decreased the inflammation response in the injured fibroblasts

**Figure 7:**
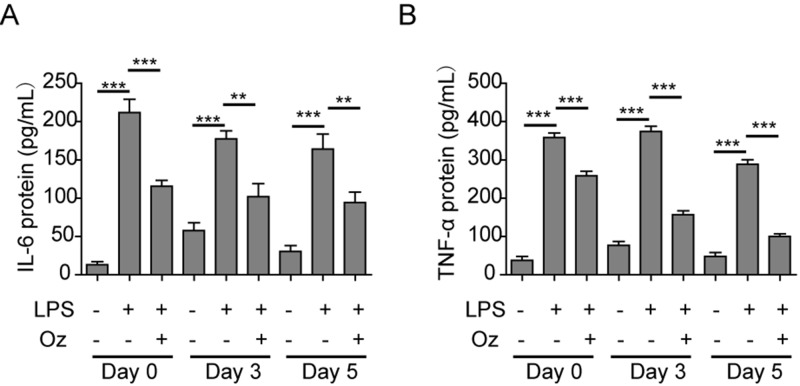
Ozone oil inhibited the inflammation of the injured fibroblasts (**A**) Ozone oil decreased the IL-6 protein level after the injured fibroblasts were treated with LPS. (**B**) Ozone oil decreased the TNF-α protein level after the injured fibroblasts were treated with LPS. Error bars represent the mean ± S.D.; **P<0.01 and ***P<0.001.

OT has been widely used for the anti-inflammatory therapy and wound healing. And it has been demonstrated that immune response plays important roles in the process of wound healing. Thus, we wanted to know if the immune response was also regulated by ozone oil during the promotion of wound healing. We treated the injured fibroblasts isolated for 0, 3, and 5 days postinjury and treated them with LPS, which could induce the immune response, together with or without ozone oil. After 1 day, we examined the expression of inflammation factors, TNF-α and IL-6, by CBA. Indeed, we found that LPS significantly induced the immune response with the up-regulation of TNF-α and IL-6, while ozone oil dramatically repressed the increase in TNF-α and IL-6 (Figure 7A,B). These data demonstrated that ozone oil could suppress the inflammation during promotion of the wound healing.

## Discussion

The injury can elicit many biological processes to promote the wound repair while the failure of this wound healing will lead to severe complications or even mortality [1,2]. The immune response is activated by injury to remove the dead cells and tissues and prevent the infection, while the persistently activated immune response also impairs the progression of wound healing [2,5,42]. Previous reports have found fibroblasts which are attracted by the wound play important roles in wound healing. Fibroblasts can secrete factors to promote angiogenesis and differentiate into myofibroblasts to form the mature scar [12,14]. Thus the studies of fibroblasts in wound healing may provide new therapeutic targets for curing the injury independent of the immune systems. Furthermore, since OT is widely applied for the anti-inflammatory therapy, we wanted to explore the function of ozone oil on the migration of fibroblasts during wound healing. And, we found that ozone oil can promote the wound healing by increasing the activation and migration of fibroblasts via PI3K/Akt/mTOR signaling pathway.

OT has been recognized as an efficient treatment for tissue repair [21-23]. OT can inactivate bacteria via disrupting their cell envelopes, inhibit fungal growth, and damage the capsid of virus. OT also increases the glycolysis rate of erythrocytes which leads to the increase in oxygen released into tissues. OT also stimulates the production of prostacyclin and the enzymes which work as cell wall protectors and free radical scavengers. Also OT is applied for tissue repair which may be through inducing the production of different growth factors VEGF, TGF-β, and PDGF. In our study, we found that ozone oil can further accelerate the rate of wound healing compared with the untreated group (Figure 1). Also, we found the treatment of ozone oil activated fibroblasts through increasing the critical genes (*collagen-I, α-SMA*, and *TGF-β1*) for fibroblasts (Figure 2). These results demonstrated that fibroblasts activities were gradually enhanced during the initial stage of new tissue formation and ozone oil promoted the wound healing via regulating the fibroblast functions.

As the tissue injury will attract the fibroblast to the injury site to repair the wound, the migration of fibroblasts is important for tissue repair [7,8,37]. Here, we found that ozone oil promoted the migration of fibroblasts which suggested the cure effect of ozone oil on wound healing through increasing the fibroblast migration (Figure 3). Furthermore, the EMT process in fibroblasts also plays important roles in tissue repair. So, we examined if the treatment of ozone oil can promote the EMT process in fibroblasts. We found that ozone oil promoted the up-regulation of critical proteins (fibronectin, vimentin, N-cadherin) and the reduction in epithelial protein (E-cadherin) in EMT (Figure 4). As IGFBPs play important roles in the EMT process, we examined the expression of these genes and found that their expression was further increased by ozone oil (Figure 4). These data demonstrated that ozone oil can activate the fibroblasts and promote the migration and EMT process in fibroblasts to facilitate the wound healing.

The EMT is an important cellular process during embryogenesis and plays critical roles in many diseases [25]. Previous studies have found many signaling pathways that are involved in the EMT process, such as TGF-β, EGF, TNF-α, IL-6, and so on [43-45]. Recent studies have shown that PI3K/Akt/mTOR signaling pathway plays important roles in EMT process [46-49]. For example, *miR-206* inhibits HGF-induced EMT process and angiogenesis via PI3K/Akt/mTOR signaling pathway in non-small-cell lung cancer [46].TGF-β2 induces the EMT process via activation of PI3K/Akt/mTOR signaling pathway in cultured human lens epithelial cells [47]. In hepatocellular carcinoma, TRAF4 regulates migration, invasion, and the EMT process via PI3K/Akt/mTOR signaling pathway [48]. So, we examined whether the regulation of ozone oil on EMT processes was through affecting the PI3K/Akt/mTOR signaling pathway. We found that the treatment of ozone oil significantly increased the activation of PI3K/Akt/mTOR signaling pathway compared with the untreated control group. The level of p-PI3K, p-Akt, and p-mTOR was gradually increased as the process of wound healing which was further increased by ozone oil (Figure 5). Importantly, we found inhibition of PI3K with LY294002 reversed the effect of ozone oil on the EMT process, which suggested that PI3K/Akt/mTOR signaling pathway is necessary for the wound healing (Figure 6). These data suggested that ozone oil promoted the EMT process via accelerating the activation of PI3K/Akt/mTOR signaling pathway to facilitate the wound healing. In future, the drugs which target PI3K/Akt/mTOR signaling pathway may provide new hope for the treatment of injury.

Inflammation is the initial step after injury and plays important roles in the process of wound healing, while the continuous inflammation will impair the wound healing process. So inhibition of the inflammation during wound healing is also critical. We found that ozone oil significantly suppressed the inflammation of the injured fibroblasts which treated with LPS (Figure 7). These results demonstrated that ozone oil can facilitate the wound healing via inhibiting the inflammation instead of promoting the function of fibroblasts and EMT.

In conclusion, we found that ozone oil can promote the wound healing via PI3K/Akt/mTOR signaling pathway. Mechanistically, we found that ozone oil can activate the fibroblasts and promote the migration of fibroblasts. Furthermore, ozone oil can further increase the EMT process in fibroblasts via up-regulating the important proteins for EMT process. Importantly, we found that PI3K/Akt/mTOR signaling pathway was involved in the regulation of ozone oil on the EMT process and wound healing. Our studies illustrate the cellular mechanisms for the treatment of ozone oil on wound healing, which will provide new insights and therapeutic targets for the treatment of tissue injury.

## Abbreviations

Akt: protein kinase B
α-SMA: α-smooth muscle actin
CBA: cytometric bead array
DMEM: dulbecco’s modified eagle medium
EGF: epidermal growth factor
EMT: epithelial–mesenchymal transition
GAPDH: glyceraldehyde-3-phosphate dehydrogenase
HGF: hepatocyte growth factor
IGFBP: insulin-like growth factor binding protein
IGF: insulin-like growth factor
IL-6: interleukin-6
LPS: lipopolysaccharid
MAPK: mitogen-activated protein kinase
MMP: matrix metallopeptidase
mTOR: mammalian target of rapamycin
OT: ozone therapy
PDGF: platelet-derived growth factor
PI3K: phosphatidylinositol 3-kinase
RIPA: radio-immunoprecipitation assay
TGF-β: transforming growth factor-β
TNF-α: tumor necrosis factor-α
TRAF4: tumor necrosis factor receptor-associated factor 4
VEGF: vascular endothelial growth factor
